# A Selective Deficit in Phonetic Recalibration by Text in Developmental Dyslexia

**DOI:** 10.3389/fpsyg.2018.00710

**Published:** 2018-05-15

**Authors:** Mirjam Keetels, Milene Bonte, Jean Vroomen

**Affiliations:** ^1^Cognitive Neuropsychology Laboratory, Department of Cognitive Neuropsychology, Tilburg University, Tilburg, Netherlands; ^2^Maastricht Brain Imaging Center, Department Cognitive Neuroscience, Faculty of Psychology and Neuroscience, Maastricht University, Maastricht, Netherlands

**Keywords:** phonetic recalibration, orthographic information, dyslexia, letters, speech perception

## Abstract

Upon hearing an ambiguous speech sound, listeners may adjust their perceptual interpretation of the speech input in accordance with contextual information, like accompanying text or lipread speech (i.e., phonetic recalibration; Bertelson et al., 2003). As developmental dyslexia (DD) has been associated with reduced integration of text and speech sounds, we investigated whether this deficit becomes manifest when text is used to induce this type of audiovisual learning. Adults with DD and normal readers were exposed to ambiguous consonants halfway between /aba/ and /ada/ together with text or lipread speech. After this audiovisual exposure phase, they categorized auditory-only ambiguous test sounds. Results showed that individuals with DD, unlike normal readers, did not use text to recalibrate their phoneme categories, whereas their recalibration by lipread speech was spared. Individuals with DD demonstrated similar deficits when ambiguous vowels (halfway between /wIt/ and /wet/) were recalibrated by text. These findings indicate that DD is related to a specific letter-speech sound association deficit that extends over phoneme classes (vowels and consonants), but – as lipreading was spared – does not extend to a more general audio–visual integration deficit. In particular, these results highlight diminished reading-related audiovisual learning in addition to the commonly reported phonological problems in developmental dyslexia.

## Introduction

Children learn to associate graphemes with speech sounds during reading acquisition. The automatic coupling of graphemes with speech is crucial to become a fluent reader in an alphabetic script. Although most children successfully master these skills, individuals with developmental dyslexia (DD) experience difficulties in reading and spelling despite adequate intelligence and intact sensory abilities (Lyon et al., 2003). Mounting evidence suggests that individuals with DD show deficits in grapheme-phoneme or letter-speech sound associations (Blau et al., 2009, 2010; Froyen et al., 2011; McNorgan et al., 2013; Zaric et al., 2014), next to commonly observed phonological processing difficulties (Snowling, 2000; Ramus, 2003; Blomert, 2011).

Blau et al. (2009) were the first to demonstrate these letter-speech sound integration impairments in a functional magnetic resonance imaging (fMRI) study. Adult dyslexic and fluent age-matched readers were presented with letters and speech sounds either in isolation (visual or auditory) or combined (congruent or incongruent). As in earlier studies, fluent readers showed enhanced superior temporal gyrus activation for congruent letter-speech sound pairs as compared to incongruent pairs (Van Atteveldt et al., 2004) indicating automatic detection of letter-speech congruencies. Blau et al. (2009), though, did not find such congruency effect for adult dyslexic readers, indicating reduced letter-speech sound integration (see also Blau et al., 2010 for similar findings in dyslexic children).

Studies using electroencaphalogram (EEG) further investigated the neural time-course of letter-speech integration deficits in individuals with DD. These studies have typically used an audiovisual variant of the oddball paradigm. In the classical oddball paradigm a mismatch negativity (MMN) response is evoked between 100 and 250 ms after the onset of a deviating sound stimulus that is presented in a sequence of repeating standard stimuli (see Näätänen et al., 2007 for a review). By employing an audiovisual oddball paradigm Froyen et al. (2008) demonstrated that normal readers show an enhanced MMN response to a deviant speech sound /o/ in a stream of standard speech sounds /a/ when both the standard and deviant sounds are presented together with the letter ‘a’ (as compared to the MMN in an auditory-only condition without letter stimuli). This enhanced audiovisual MMN indicates that in fluently reading adults, letters and speech sound are integrated early and automatically. Furthermore, these audiovisual effects have been shown to gradually appear in typically reading children after several years of reading instruction (Froyen et al., 2009; Zaric et al., 2014), whereas these effects are reduced or absent in children with dyslexia (Froyen et al., 2011; Zaric et al., 2014). Both reading-related audiovisual effects in typically reading children and their reduction in dyslexia have further been reported in EEG and fMRI studies using other paradigms and different types of stimuli including individual letters/speech sounds, syllables or words (McNorgan et al., 2013; Mittag et al., 2013; Kronschnabel et al., 2014; Zaric et al., 2014; Moll et al., 2016). (Though, for contradictory results see Nash et al., 2017).

A key question in current research on dyslexia involves the domain-specificity of this audiovisual deficit. Is it restricted to a specific deficit of matching graphemes with phonemes, or is it a more general deficit in the integration of audiovisual information (Hahn et al., 2014). At this point, findings in the literature are contradictory. Some studies suggest that individuals with DD have problems with more general audio–visual integration processes. For example, in a reaction time experiment, Harrar et al. (2014) showed that individuals with DD have problems with multisensory integration of simple non-linguistic stimuli, which would be indicative of a more general multi-sensory deficit. By using an ERP paradigm in which visual symbol patterns had to be matched with predicted sound patterns, Widmann et al. (2012) also showed that dyslexic children had difficulties to form unitary audiovisual object representations (though see Widmann et al., 2014 who showed that the gamma response in the audio–visual task is mostly due to microsacades). Other studies using lipread speech, have found mixed results. For example, Baart et al. (2012) demonstrated comparable phonetic recalibration effects by lipread speech in dyslexic and fluent readers. De Gelder and Vroomen (1998), though, reported that poor readers were also poor lipreaders, and a recent study by Van Laarhoven et al. (2018) found that both children and adults with DD have deficits in the ability to benefit from lip-read speech when speech was presented in background noise (see also Hayes et al., 2003; Ramirez and Mann, 2005). Taken together, evidence on the domain-specificity of audio–visual association deficits in DD is not consistent at this point. Furthermore, audiovisual integration has typically been studied using either text, lipread speech, *or* non-linguistic information without direct comparisons of these different types of information within the same groups.

In the present study we investigate the domain-specificity of the audiovisual processing deficit in DD by comparing the influence of written text and lipread speech on the perception of ambiguous speech sounds. If the deficit reflects a more general audiovisual deficit, impaired audiovisual processing in dyslexia should be observed with both types of information. We used lipread speech as a comparison stimulus, because, like text, it involves visual information that matches to speech sounds. Importantly, however, letters are different from lipread speech because letter-speech sound combinations are arbitrary and culturally determined and need explicit training during literacy acquisition (Liberman, 1992) and some studies even challenge the idea that written text may influence speech perception (Mitterer and Reinisch, 2015). This contrasts with the association between lipmovements and speech sounds because that does not need to be learned explicitly as there are strong biological constraints between perception and production (Kuhl and Meltzoff, 1982).

In the current study, either written text or lipread speech was presented together with ambiguous speech sounds during an exposure phase to induce phonetic recalibration. The context information (text or lipread speech) is thought to induce a shift in the perception of the ambiguous speech sound in order to reduce the intersensory conflict. This shift can then be measured as an aftereffect with subsequently presented ambiguous speech sounds. Phonetic recalibration was first demonstrated by Bertelson et al. (2003) who used an ambiguous speech sound halfway between /aba/ and /ada/ (henceforth: A?) dubbed onto the video of a face articulating either /aba/ or /ada/ (henceforth: VbA? or VdA?, where Vb = visual ‘aba’ stimulus, Vd = visual ‘ada’ stimulus, and A? = ambiguous auditory stimulus). Results showed that after exposure to an ambiguous speech sound combined with the video of a face articulating /aba/ (exposure to VbA?), an auditory-only ambiguous test sound was perceived as *more* /b/-like than after exposure to that same ambiguous sound combined with an /ada/ video (exposure to VdA?). The common interpretation is that lipread speech shifts the interpretation of the ambiguous sounds in order to reduce the intersensory conflict. This shift is thus observable as an aftereffect. Further research has also shown that this shift induced by lipread speech can be decoded in auditory cortical activity patterns (Kilian-Hutten et al., 2011).

In order to control for a simple response bias or a priming effect that reflects that a particular phoneme was heard in the previous exposure phase (e.g., participants respond /d/ simply because they heard /d/ in the foregoing exposure phase), we included, as in Bertelson et al. (2003; Experiment 2), audiovisual exposure stimuli that do not induce recalibration, namely audiovisual *congruent* stimuli with auditory *non*-ambiguous sounds: VbAb and VdAd. Nevertheless, VbAb and VdAd do not induce recalibration because there is no conflict between the heard and lipread information that induces a shift in the phoneme boundary. In previous studies, these stimuli have sometimes induced contrastive aftereffects in which the responses are in the opposite direction as the exposure stimuli (i.e., *fewer* /b/ responses after exposure to VbAb than VdAd) indicative of selective speech adaptation (Eimas and Corbit, 1973), but this effect is usually quite small as selective speech adaptation requires larger amounts of exposure stimuli (Vroomen et al., 2007).

Phonetic recalibration by lipread speech has now been replicated many times, also in other laboratories with other tokens, and other phonemes (Samuel and Kraljic, 2009; Kilian-Hutten et al., 2011; Reinisch et al., 2014; Kleinschmidt and Jaeger, 2015). Most relevant for the present study is that phonetic recalibration can also be induced by *orthographic* information (Keetels et al., 2016). As with lipread speech, normal readers thus adjust their phoneme boundary if an ambiguous speech sound is accompanied by text that specifies what the ambiguous phoneme should be. Recently, Bonte et al. (2017) replicated this text-induced recalibration effect in an fMRI-paradigm and furthermore showed that it was accompanied by subtle changes in auditory cortical activity. More specifically, their results showed that it was possible to consistently predict whether participants perceived the same ambiguous speech sounds as either /aba/ or /ada/ based on the activity patterns in the posterior superior temporal cortex (STG). This finding indicates that letter-speech sound associations can adjust the auditory cortical representation of ambiguous speech in typically reading adults.

This raises the question whether individuals with DD will have a deficit using text to induce phonetic recalibration. Of interest is that Baart et al. (2012) already found that recalibration by *lipread* speech is comparable in DD and normal readers. If indeed recalibration by lipread speech is spared in DD, we thus might expect an *orthographic-specific* deficit in the processing and integration of graphemes and phonemes rather than a more general audiovisual integration problem. In particular, this would indicate diminished reading-related audiovisual learning in DD in addition to previously reported deficits in detecting letter-speech sound (in)congruency and commonly reported phonological problems.

## Experiment 1

### Materials and Methods

#### Participants

Thirty-six students from Tilburg University participated. Eighteen of them were formally assessed and diagnosed with dyslexia, either by a remedial educationalist or psychologist (15 female; average age 20.2 ± 1.9 SD). The diagnosis was made at varying ages ranging from approximately 7–20 years. Most of them participated in a training or rehabilitation program that varied from extra reading lessons at school to remedial teaching programs at external organizations. Seven of them reported to have one or more relatives with an official dyslexia diagnosis, five reported to have no relatives with dyslexia and the others were not sure. The other eighteen participants had no diagnosis of dyslexia (13 female; average age 20.0 ± 2.0 SD) and served as a control group. Dyslexic students were invited by email and were paid for their participation and students without dyslexia participated to receive course credits. We determined our sample size based on our lab’s previous experience with the phonetic recalibration paradigm (Baart et al., 2012: 22 subjects in both the DD and Control group; Bertelson et al., 2003: 10 subjects in Experiment 2; Keetels et al., 2016: 22 subjects in both Experiments 1 and 2), which shows that inclusion of about 20 participants per subject group should give robust and significant behavioral recalibration/adaptation effects. All participants reported normal hearing and normal or corrected-to-normal vision and were fluent speakers of Dutch. They took part in the experiment individually and were unaware of the purpose of the experiment. This study was carried out in accordance with the recommendations of local ethics committee (EC-2014.38). The protocol was approved by the local ethics committee (EC-2014.38). All subjects gave written informed consent in accordance with the Declaration of Helsinki.

#### Reading Fluency Tests

Reading fluency was tested by using two Dutch standardized tests that measured single word reading for real words (‘Een-minuut-test’ or EMT, Brus and Voeten, 1997) and pseudo-words (‘De Klepel,’ Van den Bos et al., 1999). Participants had to read-out-loud as many words as possible in a certain time period (1 min for EMT, 2 min for De Klepel). For both tests, reading fluency scores were calculated by subtracting the number of mistakes from the total number of read words. As expected, the DD-group was less efficient in reading (number of correctly read real-words = 77.8 ± 3.2 SEM, pseudo-words = 75.1 ± 3.4 SEM) than the Control group (number of correctly read real-words: 101.7 ± 2.31 SEM; pseudo-words = 98.6 ± 3.5 SEM) [independent samples *t*-test: *t*(34) = 6.04, *p* < 0.001, η^2^ = 0.52 on real-words, *t*(34) = 4.83, *p* < 0.001, η^2^ = 0.41 on pseudo-words].

#### Stimuli and Materials

Participants were seated in front of a 17-inch (600 pixels × 800 pixels) CRT-monitor (100 Hz refresh rate) at a distance of approximately 60 cm. The stimuli were identical to those used in Bertelson et al. (2003). In short, we used the audiovisual recording of a male Dutch speaker pronouncing the non-words /aba/ and /ada/.

The audio was synthesized into a nine-token /aba/–/ada/ continuum (i.e., A1-A9) by changing the second formant (F2) in eight steps of 39 Mel using the ‘Praat’ speech editor (Boersma and Weenink, 1999). The offset frequency of the first vowel (before the closure) and onset frequency of the second vowel (after the closure) were 1100 Hz for /aba/ and 1678 Hz for /ada/ (see Figure 1 in Vroomen et al., 2004b). The duration of all sound files was 640 ms. From this nine-token continuum, we used the most outer tokens (A1 and A9; henceforth Ab and Ad, respectively) and the three middle tokens (A4, A5, and A6; henceforth A?-1, A? and A?+1, respectively). The audio was delivered binaurally through headphones (Sennheiser HD201). The sound volume of the stimuli was approximately 66 dB SPL when measured at 5 mm from the earphone.

Visual stimuli consisted of either the presentation of the three letters of the non-words *‘aba’* or *‘ada,’* and the video of the lip-movements of the speaker pronouncing *‘aba’* or *‘ada.’* The letters were lowercase presented in gray (RBG: 128,128,128) Arial Black Font on a dark background in the center of the screen (W: 5.5°, H: 2.5°). Visual stimulus duration was 1200 ms. When presented in combination with speech sound stimuli, letters were presented 450 ms before the sound because informal pilot testing in Keetels et al. (2016) showed that this was the most optimal interval to induce perceptual synchrony between the inner speech of the silently read letters (the internal voice that is ‘heard’ while reading) and the externally presented speech sound.

In case of the video-presentations, we used the video tracks of the audio–visual recording of the male Dutch speaker pronouncing the non-words /aba/ and /ada/. The videos showed the face of the speaker from the forehead to the chin and had a duration of 2130 ms. Videos were displayed as a string of 71 bitmaps in which each bitmap was displayed for 30 ms (including a 4 bitmap black-to-color fade-in and 5 bitmap color-to-black fade-out). The image size was 9 × 6.5 degrees (high × width) and was presented on a black background at the center of the screen.

#### Design and Procedure

**FIGURE 1:**
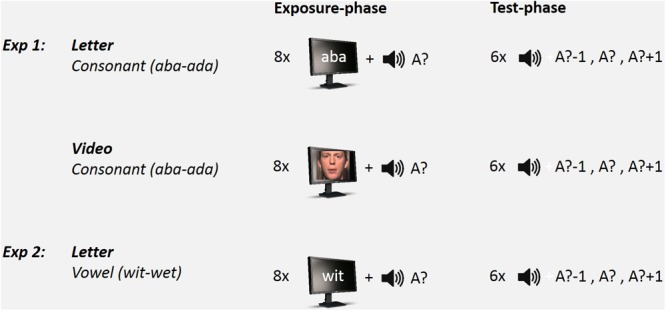
Schematic set-up of the Exposure-Test paradigm (only exposure to ambiguous speech sounds is shown). In Experiment 1, participants were exposed to 8 auditory-visual exposure stimuli followed by 6 auditory-only test trials on an ‘aba’–‘ada’ continuum. Visual stimulus type during exposure consisted of either Text (Letter) or Lipread (Video) stimuli. In Experiment 2, participants were only exposed to Text stimuli and tested on a ‘wit’–‘wet’ continuum.

Participants were repeatedly presented with Exposure-Test mini-blocks that each consisted of eight audiovisual exposures (i.e., exposure-phase) followed by six auditory-only test trials (test-phase). See **Figure 1** for a schematic set-up of the Exposure-Test mini-block design. In the Exposure phase, three within-subjects factors were varied: Exposure-type (Letter or Video), Exposure-sound (Ambiguous or Non-ambiguous) and Exposure-token (‘aba’ or ‘ada’). The exposure stimuli thus either contained letters or videos as visual stimuli in which either the ambiguous speech sound was combined with ‘aba’ or ‘ada’ (VbA? or VdA?), or the non-ambiguous speech sound in combination with congruent letters or video (VbAb or VdAd). The inter-stimulus interval (ISI) between subsequent exposure sound stimuli was 800 ms. The audiovisual exposure phase was followed (after 1500 ms) by six auditory-only test trials. Test-sounds were the most ambiguous token on the continuum (A?), its more ‘aba-like’ neighbor (A?-1), and the more ‘ada-like’ neighbor on the continuum (A?+1). The three test-sounds (A?-1; A?; A?+1) were presented twice in random order. The participant’s task was to indicate whether the test sound was more /aba/ or /ada/-like by pressing a corresponding key on a response box. The inter-trial interval (ITI) was 1250 ms.

Each participant completed 80 Exposure-Test mini-blocks in which each of the 8 exposure conditions (2 Exposure-type × 2 Exposure-sound × 2 Exposure-token) was presented 10 times (in order to collect 20 repetitions of each Test-sound per exposure condition). There was a short pause after each 16 mini-blocks. The audiovisual exposure conditions varied randomly between mini-blocks. Total testing lasted ∼60 min.

### Results

**FIGURE 2:**
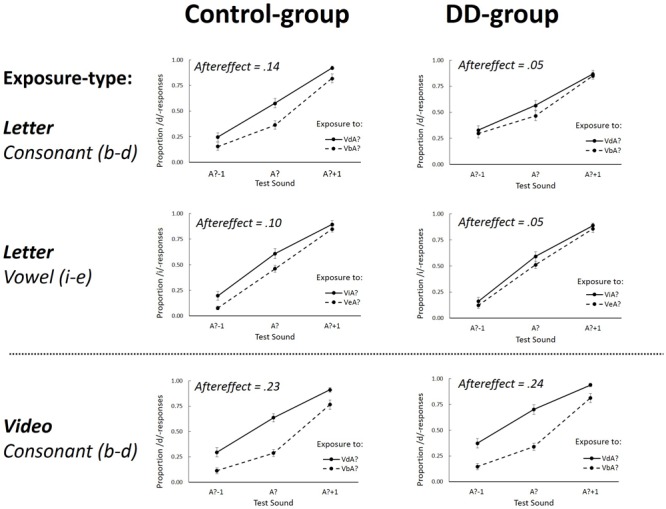
The proportion of /d/ (Experiment 1, Consonant) or /i/-responses (Experiment 2, Vowel) as a function of the three different Test-sounds (A?–1; A? and A?+1) after ambiguous Exposure-sounds. Graphs separately depict Letter (upper four graphs) and Video (lower two graphs) Exposure-types for the Control group (left graphs) and DD group (right graphs). Aftereffects represents the overall difference between the two Exposure-tokens (VdA? – VbA? for consonants, and ViA?–VeA? for vowels). Error bars represent the standard errors of the mean.

**FIGURE 3:**
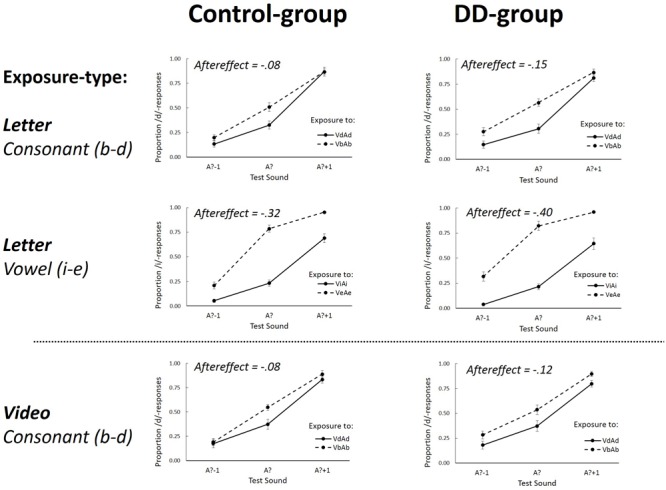
The proportion /d/ (Experiment 1, Consonant) or /i/-responses (Experiment 2, Vowel) as a function of the three different Test-sounds (A?–1; A? and A?+1) after non-ambiguous Exposure-sounds. Graphs separately depict Letter (upper four graphs) and Video (lower two graphs) Exposure-types for the Control group (left graphs) and DD group (right graphs). Aftereffects represents the overall difference between the two Exposure-tokens (VdAd – VbAb for the consonants, and ViAi–VeAe for the vowels). Error bars represent the standard errors of the mean.

The results of the ambiguous and non-ambiguous exposure sounds were analyzed separately because previous studies have demonstrated that different mechanisms underlie phonetic recalibration (induced by intersensory conflict) and selective speech adaptation (mainly depending on the acoustic nature of the exposure stimuli) (Eimas and Corbit, 1973; Samuel, 1986; Vroomen et al., 2004a; Samuel and Lieblich, 2014). **Figures 2** and **3** display the group-averaged proportions of /d/-responses of the test sounds after exposure to ambiguous and non-ambiguous sounds, respectively. As expected, after exposure to ambiguous sounds, there were *more* /d/ responses after exposure to VdA? than after VbA? (indicative of phonetic recalibration), whereas for non-ambiguous exposure, there were *fewer* /d/ responses after exposure to VdAd than after VbAb (indicative of selective speech adaptation). The individual proportion of /d/-responses on the auditory-only test-trials was calculated for each combination of Exposure-type (Letter or Video), Exposure-sound (Ambiguous or Non-ambiguous), Exposure-token (Vb or Vd), and Test-sound (A?-1; A?; A?+1).

#### Aftereffects Following Exposure to Ambiguous Sounds (Recalibration)

A repeated measures ANOVA with within-subjects factors Exposure-type (Letter or Video), Exposure-token (Vb or Vd), and Test-sound (A?-1; A?; A?+1) and between-subjects factor Dyslexia (DD or Control-group) was performed on the log-odds transformed proportions of /d/-responses on the test trials. The log-odds transformation was performed to meet assumptions of distribution normality. In cases in which Mauchly’s test indicated that the assumption of sphericity was violated, degrees of freedom were corrected using Greenhouse-Geisser estimates of sphericity.

The analysis showed a main effect of Exposure-token [*F*(1,34) = 79.96, *p* < 0.001, $\eta_{p}^{2}$ = 0.70] which interacted with Exposure-type [*F*(1,35) = 36.49, *p* < 0.001, $\eta_{p}^{2}$ = 0.52] indicative of differences between letter- and lipread-induced aftereffects (i.e., the difference between Vb and Vd Exposure-tokens). Important for the present study, this interaction was different for the DD and Control group [Exposure-token × Exposure-type × Dyslexia: *F*(1,34) = 3.58, *p* = 0.034, one-tailed, $\eta_{p}^{2}$ = 0.10] and will be further examined by *post hoc t*-tests (described below).

The ANOVA showed a main effect of Test-sound [*F*(2,68) = 212.23, *p* < 0.001, Greenhouse-Geisser corrected, $\eta_{p}^{2}$ = 0.86] which interacted with Exposure-token [*F*(2,68) = 4.82, *p* = 0.011, $\eta_{p}^{2}$ = 0.12]. Numerical comparison of the means shows overall larger aftereffects at the most ambiguous Test-sound. Also, a three-way interaction between Test-sound, Exposure-type and Dyslexia was found [*F*(2,68) = 5.00, *p* = 0.01, $\eta_{p}^{2}$ = 0.13] possibly reflecting a somewhat less steep function of Test-sound for the DD group when exposed to letters as compared to lipread exposure. The four-way interaction was not significant [*F*(2,68) = 0.112, *p* = 0.89, $\eta_{p}^{2}$ = 0.003]. None of the other effects were significant (all *p*-values > 0.17).

In order to further explore the theoretically important three-way interaction between Exposure-token, Exposure-type and Dyslexia, data were pooled over Test-sound (A?-1; A?; A?+1) and aftereffects were computed as in previous studies by subtracting the proportion of /d/ responses after exposure to VbA? from VdA? (Van Linden and Vroomen, 2007; Keetels et al., 2015, 2016). Aftereffects indicative of recalibration should then have a positive sign.

##### Letter-induced aftereffects

After exposure to ambiguous sounds combined with letter-stimuli, aftereffects were 0.05 and 0.14 for the DD and Control group, respectively. An independent samples *t*-test showed that the effect was stronger for the Control group than the DD group [*t*(34) = 2.35, *p* = 0.013 one-tailed, η^2^ = 0.14 because there was a clear prediction that DD should have smaller letter-induced recalibration effects]. Two one-sample *t*-tests were conducted using Bonferroni corrected alpha levels of 0.025 (0.05/2) per test and showed that the aftereffects were significantly different from zero for the Control group [*t*(17) = 4.35; *p* < 0.001, η^2^ = 0.53], but not for the DD group [*t*(17) = 1.51; *p* = 0.15, η^2^ = 0.12]. Dyslexic readers thus had no letter-induced recalibration effect whereas the fluent readers did.

##### Lipread-induced aftereffects

After exposure to ambiguous sounds combined with lipread speech, aftereffects were 0.24 and 0.23 for the DD and Control group, respectively. Separate one-sample *t*-tests using Bonferroni corrected alpha levels of.025 (0.05/2) tested the aftereffects against zero and showed that both groups had lipread-induced aftereffects [DD group: *t*(17) = 6.38, *p* < 0.001, η^2^ = 0.71; Control group: *t*(17) = 8.12, *p* < 0.001, η^2^ = 0.80]. Furthermore, an independent samples *t*-test showed that these effects were not different in size [*t*(34) = 0.014, *p* = 0.98, η^2^ < 0.001]. Dyslexic and fluent readers thus both had lipread-induced recalibration with comparable magnitude.

##### Lipread vs. letter-induced aftereffects

Two paired-sample *t*-tests using Bonferroni corrected alpha levels of 0.025 (0.05/2) compared the lipread and letter-induced aftereffects for both the DD and Control group. In both groups the letter-induced aftereffects were significantly smaller than the lipread-induced aftereffects [DD group: *t*(17) = 3.07, *p* < 0.01, η^2^ = 0.58; Control group: *t*(17) = 4.85, *p* < 0.001, η^2^ = 0.43].

##### Correlation between reading fluency scores and aftereffects

No significant correlations were found between the word or pseudo-word reading fluency scores and the letter-induced aftereffects (real words: *r* = 0.13, *p* = 0.44; pseudo-words: *r* = 0.26, *p* = 0.12) or the lipread aftereffects (real words: *r* = 0.20, *p* = 0.23; pseudo-words: *r* = 0.14, *p* = 0.42). Though, when correlating the reading scores with the difference between the lipread and letter induced aftereffects, a trend was found (real-words, *r* = 0.28, *p* = 0.10; pseudo-words: *r* = 0.32, *p* = 0.06) indicating a trend toward a bigger difference between lipread and letter-induced aftereffects when reading fluency was less good. The absence of significant effects might be explained by the overlap in reading scores between the groups (Controls range from 55 to 113 on the pseudo-word reading, while DDs range from 57 to 106 on pseudo-word reading). These reading scores also show that our dyslexic group consisted of compensated dyslexic adults, who were, however, all formally diagnosed with dyslexia, while participants in the control group were not.

#### Aftereffects Following Exposure to Non-ambiguous Sounds (Selective Speech Adaptation)

A repeated measures ANOVA on the data of the non-ambiguous exposure-sound trials showed a main effect of Exposure-token [*F*(1,34) = 28.10, *p* < 0.001, $\eta_{p}^{2}$ = 0.45] indicative of selective speech adaptation effects (i.e., the difference between VbAb and VdAd exposure). This effect did not interact with Exposure-type [*F*(1,34) = 0.021, *p* = 0.88, $\eta_{p}^{2}$ = 0.001], nor with Dyslexia [*F*(1,34) = 0.37, *p* = 0.55, $\eta_{p}^{2}$ = 0.011], and also no three-way interaction between these factors was found [*F*(1,34) = 0.24, *p* = 0.63, $\eta_{p}^{2}$ = 0.007]. These findings thus indicate that selective speech adaptation effects after letter and lipread exposure were not different for the DD and Control group (aftereffects after letter exposure were -0.15 and -0.08 for the DD and Control group, respectively, and aftereffects after lipread exposure were -0.12 for the DD and -0.08 for the Control group).

The analysis also showed a main effect of Test-Sound [*F*(2,68) = 261.10, *p* < 0.001, $\eta_{p}^{2}$ = 0.89] which did not interact with Dyslexia [*F*(2,68) = 2.09, *p* = 0.13, $\eta_{p}^{2}$ = 0.06]. Furthermore, an interaction between Test-sound and Exposure-token was found [*F*(2,68) = 3.19, *p* = 0.05, $\eta_{p}^{2}$ = 0.09] which interacted with Exposure-type [*F*(2,68) = 5.31, *p* = 0.007, $\eta_{p}^{2}$ = 0.14], showing that aftereffects were somewhat bigger for the most ambiguous Test-sound after letter exposure, while this was not the case for lipread exposure. None of the other effects were significant (all *p*-values > 0.13).

Taken together, Experiment 1 demonstrates that dyslexic readers had difficulties using text to recalibrate their /b-d/ phoneme boundary, whereas recalibration by lipread speech was as in normal readers. This is indicative of a rather specific deficit in the processing and integration of graphemes and phonemes in DD, but not of a more general problem in audiovisual integration. In Experiment 2 we investigated whether this deficit is replicated when vowels are used instead of consonants.

## Experiment 2

In Experiment 2, we investigated whether vowels, rather than consonants, can be recalibrated by text. It has been argued that dyslexic readers may have specific difficulties in the processing of stop consonants because the relevant acoustic cues that discriminate stop consonants from each other are short and easily masked by other acoustic information (Tallal, 1980). For this reason it is important to assess whether recalibration by text is spared if vowels instead of consonants are used. To do so, we created an ambiguous vowel halfway between /I/ and /e/ and embedded it in a CVC context of /w?t/. This sound was then accompanied by the letters ‘wit’ or ‘wet,’ that are both high-frequency words in Dutch (meaning ‘white’ and ‘law,’ respectively). Here we chose to use real words instead of pseudo-words in order to avoid any subtle differences in reading of non-word stimuli due to commonly reported phonological processing difficulties (Yap and Vanderleij, 1993; Snowling, 1995; Taroyan and Nicolson, 2009). The question was whether DD would still have deficits using written high-frequency real-words, to induce recalibration of the ambiguous vowel.

### Materials and Methods

Experimental procedures were as in Experiment 1 with the following changes.

#### Participants

Thirty-seven students from Tilburg University participated and received course credits or were paid for their participation. Nineteen of them formally diagnosed with dyslexia (average age = 21.2 ± 2.22 SD; 11 also participated in Experiment 1) and the other eighteen had no diagnosis of dyslexia and served as a control group (average age 19.3 ± 2.2 SD; two also participated in Experiment 1).

#### Reading Fluency Tests

Numerical comparison showed that individuals with DD were less efficient readers (number of correctly read real-words = 75.9 ± 3.7 SEM, pseudo-words = 72.5 ± 4.1 SEM) than the Control group (number of correctly read real-words: 92.7 ± 3.4 SEM; pseudo-words = 101.6 ± 1.5 SEM), a finding that was confirmed by two independent samples *t*-tests with Bonferroni corrected alpha levels of 0.025 (0.05/2) [*t*(35) = 3.35, *p* = 0.002, η^2^ = 0.24 for real-words, *t*(35) = 6.44, *p* < 0.001, η^2^ = 0.54 for pseudo-words].

#### Stimuli and Materials

For the auditory stimuli, we used the audio tracks of a recording of a male Dutch speaker pronouncing the words /wet/ and /wIt/. The audio was synthesized into a 19-token /wet/–/wIt/ continuum (i.e., A1–A19) created with Tandem-STRAIGHT (Kawahara et al., 2008) by changing the spectrum and fundamental frequency of the individual tokens. The duration of all sound files was 595 ms. From this nineteen-token continuum, we used the most outer tokens (A1 and A19; henceforth Ae and Ai, respectively), and three tokens from the middle of the continuum (A8, A10, and A12, henceforth A?-1, A? and A?+1, respectively). These three middle tokens were chosen based on pilot-testing showing a comparable categorization curve as the middle tokens of the /aba/-/ada/ continuum of Experiment 1. The audio was delivered binaurally through headphones (Sennheiser HD201) in which the sound volume of the stimuli was approximately 64 dB SPL when measured at 5 mm from the earphone.

Visual stimuli consisted of the presentation of the three letters of the Dutch words ‘wit’ and ‘wet’. As in Experiment 1, the letters were gray on a dark background with a duration of 1200 ms and presented 450 ms before the onset of the audio.

#### Design and Procedure

As in Experiment 1, Exposure-Test mini-blocks were presented in which Exposure-sound (Ambiguous or Non-ambiguous) and Exposure-token (‘wit’ or ‘wet’) and Test-sound (A?-1; A?; A?+1) were varied. The participant’s task was to indicate whether the test sound was more like /wIt/ or /wet/. Each participant completed 40 Exposure-Test mini-blocks in which each of the 4 exposure conditions [Exposure-sound (Ambiguous/Non-ambiguous) × Exposure-token (‘wit’/’wet’)] was presented 10 times.

### Results

Analyses were performed on the log odds transformations of the individual proportion of /i/-responses (i.e., ‘wit’-responses) on the auditory-only test-trials (see **Figures 2** and **3**). As in Experiment 1, in cases in which Mauchly’s test indicated that the assumption of sphericity was violated, degrees of freedom were corrected using Greenhouse-Geisser estimates of sphericity.

#### Aftereffects Following Exposure to Ambiguous Sounds (Recalibration)

A repeated measures ANOVA with within-subjects factors Exposure-token (Vi or Ve) and Test-sound (A?-1; A?; A?+1) and between-subjects factor Dyslexia (DD or Control group) was performed on the log-odds transformed proportions of /i/-responses to the test sounds. A main effect of Test-sound [*F*(2,70) = 348.474, *p* < 0.001, $\eta_{p}^{2}$ = 0.91] was found, indicative of an overall larger number of /i/-responses for Test-sounds that were more /i/-like. Importantly, also an effect of Exposure-token [*F*(1,35) = 12.49, *p* < 0.001, $\eta_{p}^{2}$ = 0.26] was found, indicative of phonetic recalibration, and this effect interacted with Dyslexia [*F*(1,35) = 3.70, *p* = 0.032, one-tailed, $\eta_{p}^{2}$ = 0.10] showing a significant group difference in phonetic recalibration with vowels. None of the other effects were significant (all *p*-values > 0.61).

To measure aftereffects, data were pooled, as before, over the three Test-sounds (A?-1; A?; A?+1) and the difference was computed between exposure to ViA? and VeA?. After exposure to ambiguous sounds, aftereffects were 0.05 and 0.10 for the DD and Control group, respectively. Two one-sample *t*-tests were conducted using Bonferroni corrected alpha levels of 0.025 (0.05/2) and showed that the effect was significantly different from zero for the Control group [*t*(17) = 3.80; *p* = 0.001, η^2^= 0.46], but not for the DD group [*t*(18) = 1.16; *p* = 0.26, η^2^= 0.07]. In line with the data of Experiment 1, dyslexic readers thus showed no letter-induced phonetic recalibration while the fluent readers did.

#### Aftereffects Following Exposure to Non-ambiguous Sounds (Selective Speech Adaptation)

A repeated measures ANOVA was also performed on the log-odds transformed proportion of /i/-responses after exposure to non-ambiguous sounds. This analysis showed a main effect of Test-sound [*F*(2,70) = 489.82, *p* < 0.001, $\eta_{p}^{2}$ = 0.93] which interacted with Exposure-token [*F*(2,70) = 13.78, *p* < 0.001, $\eta_{p}^{2}$ = 0.28] showing that aftereffects were strongest at the most ambiguous test-sound. A main effect of Exposure-token was found [*F*(1,35) = 379.97, *p* < 0.001, $\eta_{p}^{2}$ = 0.92] indicative of selective speech adaptation (i.e., ViAi and VeAe difference). This effect interacted with Dyslexia [*F*(1,35) = 4.28, *p* = 0.046, $\eta_{p}^{2}$ = 0.11] due to slightly more negative aftereffects in dyslexics (-0.40 and -0.32 for the DD and Control group, respectively). None of the other effects were significant (all *p*-values > 0.68). *Post hoc* one-sample *t*-tests showed that aftereffects were significantly smaller than zero in both the DD [*t*(18) = 14.22, *p* < 0.001, η^2^= 0.92] and the Control group [*t*(17) = 13.53, *p* < 0.001, η^2^= 0.92].

## Discussion

As developmental dyslexia has been associated with reduced integration of text and speech sounds, we investigated whether this deficit becomes manifest when text is used to induce phonetic recalibration. More specifically, we investigated whether dyslexic readers use orthographic information to recalibrate their phoneme boundary and compare this to their ability to use lipread speech for recalibration. In Experiment 1, adults with DD had no text-induced recalibration for a /b-d/ phoneme boundary, whereas lipread-induced recalibration was normal. In Experiment 2, the same absence of text-induced recalibration was found for an /e-I/ boundary. Together, these results demonstrate that dyslexic readers do not use disambiguating orthographic information to adjust their phoneme boundaries in a comparable way as fluent readers do.

Importantly, dyslexics’ recalibration by lipread speech was as in normal readers. This is in line with Baart et al. (2012) showing that dyslexic and fluent readers have comparable lipread recalibration effects. Together, these data speak to the question whether deficits in grapheme-phoneme association in DD are specific for visual orthographic information, or are the result of a more general auditory-visual association deficit (Blomert, 2011; Hahn et al., 2014). Our data clearly suggest that dyslexic readers have a specific orthographic integration deficit. Further research is needed, though, to address this question from a broader context. In particular, others have found that DD might be associated with more general audio–visual integration processes. For example, Harrar et al. (2014) showed that dyslexics have problems with multisensory integration of simple non-linguistic stimuli, Francisco et al. (2017) showed a correlation between reading errors and audiovisual temporal sensitivity for speech and non-speech stimuli, and Widmann et al. (2012) showed that dyslexic children did not integrate visual symbolic and auditory sensory information into a unitary audiovisual object representation (though see Widmann et al., 2014). Of relevance for the present study, it remains to be examined whether individuals with DD might have more subtle integration problems with auditory and lipread speech than we could observe here (De Gelder and Vroomen, 1991). For example, it is conceivable that recalibration for lipreading was at ceiling in both groups, but that deficits in lipreading in DD would become visible if the lipread stimuli were more varied and more difficult than the relatively easy to lipread /b-d/ contrast. It might also be the case that deficits in lipreading in DD are less noticeable in these repetitive listening conditions and become more evident in more challenging listening conditions like presentations of speech in noise. This would be in line with other studies showing that adults and children with DD gain less from lipreading when speech is presented in noise (Hayes et al., 2003; Ramirez and Mann, 2005; Van Laarhoven et al., 2018).

In the present study we found that individuals with DD show intact phonetic recalibration when it was induced by lipread information, but not when induced by text. This raises the question whether dyslexics might also show deficits in another well-studied form of speech recalibration, namely phonetic recalibration driven by lexical information. Lexical recalibration was first demonstrated by Norris et al. (2003) and is a form of phonetic recalibration in which the lexical context of a spoken word provides the disambiguating information for a phonetically ambiguous sound. For example, a speech sound halfway between /f/ and /s/ is heard as /f/ when embedded in the Dutch word *witlof* (i.e., chicory) but as /s/ when embedded in *naaldbos* (i.e., pine forest). Although we are not aware of studies investigating lexical recalibration in dyslexia, Blomert et al. (2004) showed that dyslexic and normally reading children exhibit comparable context effects in speech perception at auditory, phonetic, and phonological levels of processing. Together with the presently observed absence of a general problem in audiovisual recalibration of speech, we would thus predict normal lexically driven recalibration in dyslexia. This prediction would also be in line with the typical dyslexia profile of phonological deficits combined with spared non-phonological language skills (Ramus et al., 2013). Further research is needed to examine this question.

Since the results of the present study demonstrate that dyslexic readers show specific deficits in grapheme-phoneme associations, the question arises whether training in grapheme-phoneme associations would result in less prominent reading and spelling problems in DD. In a recent study, Fraga Gonzalez et al. (2015) investigated whether an intensive 6-month letter-speech sound integration training leads to improved reading fluency in dyslexic children. The results indicated faster improvements at word reading and spelling measures in dyslexic children who followed the training in comparison to a control group of dyslexic children without training. Comparable findings were reported by Žaric et al. (2015) who further showed that deficiencies in audiovisual ERP (MMN and a late negativity) modulations that are typically shown in dyslexic readers when being presented with letter-speech sound stimuli, are reduced by letter-speech sound training. Future research might therefore investigate whether dyslexics develop orthographically induced recalibration after longer periods of training to letter-speech sound combinations.

Exposure to *non-*ambiguous speech sounds led to selective speech adaptation effects in both visual conditions (orthographic and lipread). This fits previous reports demonstrating that the origin of the aftereffects (i.e., selective speech adaptation) mostly depends on the acoustic nature of the exposure stimulus (Eimas and Corbit, 1973; Samuel, 1986; Vroomen et al., 2004a; Samuel and Lieblich, 2014) rather than on the combination of the auditory and visual stimuli, as in the case of phonetic recalibration. Given that the same auditory stimulus was used for non-ambiguous exposure in both orthographic and lipread conditions, it is not surprising that both these visual conditions induced selective speech adaptation effects. In addition, the finding that both dyslexic and normal readers showed selective speech adaptation aftereffects, suggests the absence of general speech perception deficits in dyslexia (see also Ramus, 2003; Blomert, 2011).

In Experiment 1, the audiovisual timings of lipread speech versus text (relative to the ambiguous sound) may be somewhat different from each other, but in our view this is not crucial for the interpretation of the data. With lipread speech, the sound and lip-movements were synced, but in the orthographic context, the text was presented 450 ms prior to the speech sound. At first sight, it may seem then that the text precedes the audio whereas the video does not. However, it is important to note that the videos also contain anticipatory information such that ‘b’ or ‘d’ can be lipread before the ambiguous sound is heard (although their exact timing is difficult to measure). Both the orthographic and the lipread context thus provide visual information about ‘b’ or ‘d’ before the crucial part of the sound is heard. This is in agreement with data showing that the effect of written text on the reported clarity of noise-vocoded speech is most pronounced when text is presented before (rather than after) speech, and that this effect only declines when text is presented more than 120 ms after speech onset (Sohoglu et al., 2014).

Another interesting finding that deserves further discussion is that lipread speech induced *larger* recalibration effects than text. This may seem surprising because ‘viseme’ categories for lipread speech (the class of phonemes that looks the same) do not have a one-to-one correspondence to phonemes. For example, lipread information about bilabial closure can correspond with phonemes /b/, /p/, and /m/, whereas textual information of ‘b’ unambiguously corresponds to the sound /b/. In essence, lipread speech thus contains *less* phonetic information than text, but it nevertheless induces *larger* recalibration effects. Similar observations have been made with EEG studies using an audiovisual mismatch negativity paradigm [MMN, a component of the event-related potential (ERP) reflecting pre-attentive auditory change detection] in which deviant text or lipread speech was used to induce an illusory change in a sequence of identical ambiguous sounds halfway between /aba/ and /ada/. Results showed that only deviant lipread speech induced a so-called McGurk-MMN, but not deviant text (Stekelenburg et al., 2018). Text thus appears to have weaker effects on sound processing than visual speech, also if measured at the neurophysiological level measured via EEG. It should be mentioned though that in fMRI, both lipread and text-speech sound associations do induce changes in speech perception that are measurable as subtle changes in auditory cortical activity (Kilian-Hutten et al., 2011; Bonte et al., 2017). Thus both following lipread and text-based recalibration, it is possible to retrieve participant’s perceptual interpretation of the ambiguous speech sounds from posterior auditory cortical activity patterns, indicating that both types of inducer stimuli can serve a disambiguating role in phonetic adjustments. A potential difference that may account for why lipread speech is usually more potent than text is that lipread sound-sight associations are natural and acquired early in life whereas letter-speech sound associations are culturally defined and acquired at school-age by extensive reading training (Liberman, 1992). According to this line of reasoning, it may not be that surprising that the earlier acquired lip-speech sound associations induce larger effects as compared to the later acquired text-speech sound associations. Admittedly though, further research is needed to fully elucidate the different effects that text and lipread speech have on speech sound processing.

To summarize, the present study demonstrates that, unlike fluent readers, dyslexic readers do not show orthographic induced recalibration. Together with previous findings, this suggests that individuals with DD have difficulties in learning and applying letter-speech sound associations. Since dyslexic readers did not show deficits in lipread-induced phonetic recalibration effects, these findings additionally point into the direction of auditory-visual association deficits in DD that are specific for orthographic information, rather than originating from a general auditory-visual integration deficit.

## Author Contributions

All authors contributed to the study design. Testing and data collection were performed under supervision by MK. MK performed the data analysis and drafted the manuscript. MB and JV provided critical revisions. All authors approved the final version of the manuscript for submission.

## Conflict of Interest Statement

The authors declare that the research was conducted in the absence of any commercial or financial relationships that could be construed as a potential conflict of interest. The handling Editor declared a past co-authorship with one of the authors MB.
